# Supplementary material for: Multiparametric Magnetic Resonance Imaging Correlates of Isocitrate Dehydrogenase Mutation in WHO high-Grade Astrocytomas
Source: J Pers Med. 2022 Dec 29;13(1):72. doi: 10.3390/jpm13010072 (PMC9865247; doi:10.3390/jpm13010072)
Supplement: Supplementary file 1 [file jpm-13-00072-s001.zip › jpm-1988933-supplementary.pdf]

Supplementary tables:

Table S1: VASARI imaging parameters with its modifications.

Imaging feature	VASARI parameters	Modifications
Tumor location	Frontal<br>Temporal<br>Insular<br>Occipital<br>Parietal<br>Basal ganglia<br>Thalamus<br>Brainstem<br>Cerebellum	Frontal<br>Temporal<br>Insular<br>Occipital<br>Parietal<br>Brainstem and<br>Cerebellum<br>Multiple sites
Tumor laterality	Right<br>Left<br>Central/bilateral	Right<br>Left<br>Central/bilateral
Enhancement pattern 1	No<br>Mild<br>Marked	None<br>Mild<br>Moderate<br>Severe
Enhancement pattern 2		Homogenous<br>Heterogenous
Enhancement pattern 3		Rim<br>Nodular<br>Patchy<br>Solid
Proportion enhancing	<5%<br>6-33%<br>34-67%<br>68-95%<br>>95%	None<br><25%<br>25%-50%<br>>50%
Proportion non-enhancing tumor	<5%<br>6-33%<br>34-67%<br>68-95%<br>>95%	
Eloquent brain involvement	No eloquent brain<br>Speech motor<br>Speech receptive<br>Motor<br>Vision	None<br>Wernicke's<br>Broca's<br>Motor<br>Vision<br>Multiple sites

Necrosis	<5%<br>6-33%<br>34-67%<br>68-95%<br>>95%	None<br><25%<br>25% - 50%<br>>50%
Proportion of edema	<5%<br>6-33%<br>34-67%<br>68-95%<br>>95%	None<br><tumor volume<br>Equal to tumor volume<br>>tumor volume
Hemorrhage	No<br>Yes	No<br>Yes
Cysts	Absent<br>Present	Absent<br>Present<br>Present with hemorrhage within
Multifocal/<br>Multicentric	Focal<br>Multifocal<br>Multicentric<br>Gliomatosis	Focal<br>Multifocal<br>Multicentric<br>Gliomatosis
Tumor size	<2 cm<br>2-5 cm<br>>5 cm	<2 cm<br>2-5 cm<br>>5 cm
Satellite lesions	No<br>Yes	No<br>Yes
Leptomeningeal spread		No<br>Yes
Pial invasion	No<br>Yes	
Midline shift		None<br>Mild ≤ 5mm<br>Moderate 5-10 mm<br>Severe > 10 mm
Tumor crossing midline	No.<br>Yes.	No.<br>Yes.
nCET crossing midline	No.<br>Yes.	
Calvarial remodeling	No<br>Yes	No<br>Yes-<br>Direct by tumor/<br>Indirectly by mass effect
Diffusion Restriction	Facilitated<br>Restricted	None<br>Facilitated

	Mixed<br>Indeterminate	Restricted
Ependymal invasion	No<br>Yes	No<br>Yes
Cortical involvement	No<br>Yes	
Deep white matter involvement	No<br>Yes	
Epicenter		Cortical<br>Deep white matter based
Subcortical involvement		Absent<br>Present
Margins (enhancing/ non-enhancing)	Well defined<br>Poorly defined	Well defined<br>Ill defined<br>Well-defined with areas of focal infiltration
Thickness of enhancing margin	Thin <3mm<br>Thick >3mm<br>Solid, no rim	
FLAIR/T2 mismatch		No<br>Yes
T1- FLAIR ratio	Expansive<br>Mixed<br>Infiltrative	
Perfusion		rCBV
Closeness to Sub ventricular zone (SVZ)		≤ 5mm close to SVZ<br>> 5mm away from SVZ
Dural enhancement		Absent<br>Present
Presence of vessel on T2 sequence		Absent<br>Present

Table S2: MR imaging parameters which did not significantly correlate with IDH status

Variable	Parameter	IDH-Mutated (n=19)	IDH-Wildtype (n=129)	p-value
Gender	Female	5 (26.3)	44 (34.1)	
	Male	14 (73.7)	85 (65.9)	0.680
Location	Frontal	10 (52.6)	36 (27.9)	
	Temporal	3 (15.8)	19 (14.7)	
	Insular	0 (0.0)	2 (1.6)	
	Occipital	0 (0.0)	4 (3.1)	
	Parietal	1 (5.3)	19 (14.7)	
	Brainstem/ cerebellum	0 (0.0)	2 (1.6)	
	More than one cortex	5 (26.3)	47 (36.4)	0.420
Enhancement pattern 2	Homogenous	3 (15.8)	15 (11.6)	
	Heterogenous	16 (84.2)	114 (88.4)	0.705
Enhancing proportion	None	0 (0.0)	0 (0.0)	
	< 25%	13 (68.4)	62 (48.1)	
	25- 50%	4 (21.1)	49 (38.0)	
	> 50%	2 (10.5)	18 (14.0)	0.244
Eloquent cortex	None	10 (52.6)	94 (72.9)	
	Wernicke	1 (5.3)	14 (10.9)	
	Broca	3 (15.8)	7 (5.4)	
	Motor	3 (15.8)	11 (8.5)	
	Vision	0 (0.0)	2 (1.6)	
	More than one area	2 (10.5)	1 (0.8)	0.189
Hemorrhage	No	11 (57.9)	53 (41.7)	
	Yes	8 (42.1)	74 (58.3)	0.185
Multifocal/ multicentric	Focal	16 (84.2)	104 (80.6)	
	Multifocal	3 (15.8)	19 (14.7)	
	Multicentric	0 (0.0)	5 (3.9)	
	Gliomatosis	0 (0.0)	1 (0.8)	0.820
Size	< 5 cm	4 (21.1)	51 (39.5)	
	> 5 cm	15 (78.9)	78 (60.5)	0.136
Satellite lesions	No	16 (84.2)	104 (80.6)	
	Yes	3 (15.8)	25 (19.4)	1.000
Leptomeningeal spread	No	18 (94.7)	108 (83.7)	
	Yes	1 (5.3)	21 (16.3)	0.309
Midline shift	None	1 (5.3)	19 (14.7)	
	Mild < 5 mm	8 (42.1)	38 (29.5)	
	Moderate 5- 10 mm	6 (31.6)	51 (39.5)	
	Severe > 10 mm	4 (21.1)	21 (16.3)	0.485

Tumor crossing midline	No	13 (68.4)	104 (80.6)	
	Yes	6 (31.6)	25 (19.4)	0.222
Calvarial remodeling	No	16 (84.2)	117 (90.7)	
	Directly by tumor	2 (10.5)	6 (4.7)	
	Indirectly by mass effect	1 (5.3)	6 (4.7)	0.563
Diffusion	None	12 (63.2)	49 (40.5)	
	Facilitated	0 (0.0)	2 (1.7)	
	Restricted	7 (36.8)	70 (57.9)	0.168
Ependymal invasion	No	10 (52.6)	82 (63.6)	
	Yes	9 (47.4)	47 (36.4)	0.359
Epicenter	Cortex	7 (36.8)	45 (34.9)	
	Deep white matter	12 (63.2)	84 (65.1)	1.000
	Well defined	3 (15.8)	16 (12.4)	
Margins	Ill defined	12 (63.2)	54 (41.9)	
	Well-defined with areas of focal infiltration	4 (21.1)	59 (45.7)	0.122
FLAIR-T2 mismatch	No	16 (84.2)	124 (96.1)	
	Yes	3 (15.8)	5 (3.9)	0.067
Distance from SVZ	< 5 mm close to SVZ	14 (73.7)	90 (70.3)	
	> 5 mm away from SVZ	5 (26.3)	38 (29.7)	0.975
Presence of vessel on T2	Absent	6 (31.6)	42 (32.6)	
	Present	13 (68.4)	87 (67.4)	0.932

IQR- interquartile range

SVZ- subventricular zone.
